# Impact of COVID-19 Pandemic on the Trends of *Trichomonas vaginalis* Infection in a Tertiary Hospital of Madrid, Spain

**DOI:** 10.3390/microorganisms12030620

**Published:** 2024-03-20

**Authors:** Celia Bolumburu, Vega Zamora, María Muñoz-Algarra, Maria Luisa de la Cruz Conty, José Antonio Escario, Alexandra Ibáñez-Escribano

**Affiliations:** 1 Departamento de Microbiología y Parasitología, Facultad de Farmacia, Universidad Complutense de Madrid, Plaza Ramón y Cajal s/n, 28040 Madrid, Spain; celiabol@ucm.es (C.B.); escario@ucm.es (J.A.E.); 2 Servicio de Microbiología y Parasitología Clínica, Hospital Universitario Puerta de Hierro-Majadahonda, C. Joaquín Rodrigo, 1, 28222 Madrid, Spain; vzamorafuente@gmail.com (V.Z.); maria.munoza@salud.madrid.org (M.M.-A.); 3 Departamento de Estadística e Investigación Operativa, Facultad de Medicina, Universidad Complutense de Madrid, Plaza Ramón y Cajal s/n, 28040 Madrid, Spain; ml.cruz@ucm.es

**Keywords:** *Trichomonas vaginalis*, COVID-19, prevalence, pregnancy, co-infections

## Abstract

More than one million sexually transmitted infections (STIs) occur every day, and *Trichomonas vaginalis* is responsible for more than 156 million cases each year worldwide. Nevertheless, epidemiological studies of this parasite in Europe are scarce. The aim of this study was to evaluate the impact that the COVID-19 pandemic may have had in the diagnosis and epidemiology of trichomoniasis. All available data from January 2018 to December 2021 for *T. vaginalis* isolation on gynecologic patients attending a Spanish Tertiary Hospital were analyzed. Pre-pandemic results (2018–2019) were compared to pandemic results (2020–2021). The pre-pandemic *T. vaginalis* prevalence in women was 1.15% (95% Confidence Interval, CI: 0.94–1.41), and significantly decreased in 2020–2021 (0.77%, 95% CI: 0.57–1.03; *p* = 0.025). Demographic nor clinical characteristics of women diagnosed with trichomoniasis did not statistically differ between the periods, although an increase in chlamydia co-infected patients was observed in the latest (from 8% in 2018–2019 to 19% in 2020–2021). This study has detected a decrease in the diagnosis of trichomoniasis; however, this is probably due to the increase in the healthcare pressure triggered by the pandemic. More than 75% of the cases diagnosed in 2021 occurred in the second half, which suggests that special attention should be given to the evolution in the coming years once normality has been restored in hospitals. Moreover, these results warn of the lack of routine diagnosis of trichomoniasis during pregnancy and the absence of specific protocols for possible co-infections, which could become a strategy to reduce the growing trend of STIs, including *T. vaginalis* detection, as an interesting marker of sexual risk behaviors.

## 1. Introduction

*Trichomonas vaginalis* is the etiological agent of trichomoniasis, the most common non-viral sexually transmitted infection (STI) worldwide [1]. Nevertheless, the number of reports about trichomoniasis prevalence in Europe are scarce; the last global estimation reported an increase in prevalence from 1.0% in data collected by the WHO from 2005–2012 to 1.6% between 2009–2016 in women, while in men it doubled [2,3]. In Spain, in particular, only four prevalence studies have been carried out, three of them in Granada (2011–2014) [4,5,6] and one in the Community of Madrid with clinical data from 2013–2017 [7], showing that in this country, the prevalence of trichomoniasis might be between 0.8–2.4%. However, the real prevalence in Spain is unknown, and further studies are necessary.

The fact that trichomoniasis is not considered a reportable disease explains the lack of solid data about the global and national prevalence of this parasitic infection, and could imply the underestimation of the real epidemiological rates. The less severity of symptoms compared to other STIs (e.g., HIV, syphilis, HPV, among others), the scarce public awareness associated with trichomoniasis, and the unclear socioeconomic burden of this STI explain why trichomoniasis is still not a notifiable disease today [8]. Nonetheless, the serious consequences derived from trichomoniasis demonstrate why health agencies such as the WHO have recently included this parasitic infection in their Global Health Strategies on STIs for the period of 2022–2030. Specifically, one of the targets is to reduce the total number of cases of trichomoniasis, chlamydia, syphilis, and gonorrhoea cases among people aged 15–49 from the estimated 374 million in 2020 to less than 150 million new cases in 2030 [9].

Trichomoniasis is associated with a broad range of symptoms from vaginitis to neoplastic processes, such as cervical or prostate cancer [10,11], as well as infertility [12]. Moreover, this STI can increase the probability of acquiring HIV, Human Papilloma Virus, Herpes simplex virus types 1 and 2, and other bacterial STIs (*Neisseria gonorrhoeae*, *Chlamydia trachomatis*, *Treponema pallidum*) [13,14]. Regarding pregnancy, a recent meta-analysis which included 17 studies concluded that *T. vaginalis* is associated with preterm births (OR 1.27; 95% CI 1.08–1.50), premature rupture of membranes (OR 1.87; 95% CI 1.53–2.29), and low birthweight (OR 2.12; 95% CI 1.15–3.91) [15]. Therefore, the absence of routine screening for trichomoniasis in pregnant women seems to pose an additional risk, since a potential cause of pregnancy outcomes could be detected.

On the other hand, there are only two drugs approved for treating trichomoniasis infection in Spain—metronidazole and tinidazole—while secnidazole has been newly approved by the Food and Drug Administration for treatment of this STI [16]. Nevertheless, metronidazole resistance is estimated in 4–17% of clinical isolates [17]. These drugs belong to the same chemical family (5-nitroimidazoles) and therefore have the same mode of action, making cross-resistance possible [18]. Antimicrobial resistance has arisen as one of the main challenges we face nowadays, as alerted by international organizations such as the WHO and UN [19]. Multi-drug resistance has extended to include sexually transmitted pathogens, which are becoming a serious health problem in certain patients infected with *N. gonorrhoeae*, *C. trachomatis*, *T. vaginalis*, *Mycoplasma hominis*, *M. genitalium*, or *Haemophilus ducreyi* [20].

However, in 2020, health concerns changed drastically. In January, the Center of Disease and Control from China informed that the etiological agent responsible for the pneumonia cases reported by the Wuhan Municipal Health Commission was a novel coronavirus (2019-nCoV) [21]. The outbreak was declared as a pandemic on 11 March 2020 by the WHO, and aiming to contain the spread of the virus, Spain declared the state of alarm three days after. To date, Coronavirus Disease 19 (COVID-19) has provoked more than 0.27 billion confirmed cases in the WHO European Region, and more than 121,852 deaths in Spain [22]. After SARS-CoV-2 infection, the immune system undergoes certain alterations that can persist for months after recovery [23,24]. Moreover, the psychological consequence of confinement among the general population and health professionals has become a concern [25,26]. In terms of sexual behavior, restrictive and containment policies triggered internet abuse, including an increase in pornography consumption and other behavioral changes [27,28], with a consequent impact on STI rates. Changes resulting from the COVID-19 pandemic in terms of more telemedicine visits, changes in sexual activity, the lower number of STI tests, or the greater impact on certain low-income groups that are more likely to acquire STIs [29], underscore the need to monitor the prevalence of STIs, including trichomoniasis.

Although the infection alters the immune system of patients and sexual habits and behaviors have been changing, the real impact on the prevalence of sexually transmitted infections is unknown [29]. Due to this, the main goal of this study was to determine the possible impact of the COVID-19 pandemic on the diagnosis and epidemiology of trichomoniasis in women. This was carried out by comparing the results of two periods, pre-pandemic (years 2018–2019) and pandemic (years 2020–2021), and specifically the prevalence of *T. vaginalis* and the demographic and clinical characteristics of infected patients, including pregnancy, as well as the presence of co-infection by other STIs.

## 2. Materials and Methods

### 2.1. Study Settings

A retrospective observational descriptive study was conducted, including data from 2018–2021, in Puerta de Hierro Majadahonda Hospital (Madrid, Spain), a tertiary hospital which attends near 550,000 citizens. The inclusion criteria were women diagnosed with trichomoniasis between January 2018 and December 2021, no matter its origin (i.e., Primary Care, Urgent Service, Gynecology Service covered by the Hospital), and with data included in the hospital’s medical records. The exclusion criteria were males, as *T. vaginalis* isolation was incidental, including a total of 6 cases from urethral exudates and urine samples during the study period. A total of 16,353 vaginal samples were analyzed for the presence of *T. vaginalis* (9788 between 2018–2019 and 6565 between 2020–2021). Additionally, it was investigated whether a screening for other co-infections was processed or not (*N. gonorrhoeae*, *C. trachomatis*, *T. pallidum*, and HIV) in the patients that were positive for trichomoniasis. *Mycoplasma hominis* and *Ureaplasma urealyticum* were also included in the screening, due to their clinical importance in pregnant women. 

### 2.2. Sample Collection and Culture

Vaginal swabs were collected into a sterile sampling container with Stuart medium (Deltalab Amies, Spain) and analyzed by direct microscopy. If *T. vaginalis* was not isolated, but frequent leukocytes (more than 10 leukocytes per 40× field of view) or clue cells were observed, and vaginal swabs were cultivated in Roiron medium (Maim, Spain) for 4–7 days at 37 °C. Positive samples were sent to the biomolecular laboratory and cultivated in modified trypticase-yeast extract-maltose (TYM) medium supplemented with 10% (*v*/*v*) heat-inactivated fetal bovine serum, and antibiotic solutions [7].

### 2.3. Microbial Identification

Endocervical swabs were used for *N. gonorrhoeae* and *C. trachomatis* detection (BD Universal Viral Transport-UVT) by Real Time PCR (BDMax, Beckton Dickinson, Franklin Lakes, NJ, USA), while *T. pallidum* and HIV were diagnosed in serum by automated chemiluminescence immunoassay using the Liaison^®^X (DiaSorin, Saluggia, Italy) and ADVIA Centaur^®^XP Immunoassay System (Siemens Healthineers, Forchheim, Germany). *M. hominis* and *U. urealyticum* were detected by the Mycoplasma IST2 kit (Biomérieux, Marcy l´Étoile, France). Infection was considered over colonization when ≥10,000 CCU (Colour Changing Units), according to the manufacturer’s instructions.

### 2.4. Statistical Analysis

The available information, both the prevalence and the demographic and clinical characteristics of the patients who tested positive for *T. vaginalis*, was grouped into two periods: pre-COVID-19, from January 2018 to December 2019, and COVID-19, from January 2020 to December 2021, to evaluate the impact of the COVID-19 pandemic in the epidemiology of *T. vaginalis* in women.

Demographic and clinical information of the positive patients was collected from their medical records; this information included age, nationality, whether they were pregnant at the time of diagnosis, symptomatology, inflammation parameters, and whether they had any co-infection (by sexually transmitted infection). Three groups were formed for the nationality of the patients: Spanish, Central–South American, and others (different from the two previous groups). If the patient was symptomatic, the main signs and symptoms corresponding to trichomoniasis were listed (leucorrhea, pruritus, vaginitis/vulvitis, and dysuria, bearing in mind that they could present more than one of these signs/symptoms). Regarding inflammation, patients were also grouped based on the level of leukocytes as follows: no leukocytes, low leukocytes, and frequent leukocytes [30]. Finally, the studied co-infections were *Neisseria gonorrhoeae*, *Chlamydia trachomatis*, Human Immunodeficiency Virus, *Treponema pallidum*, *Mycoplasma hominis*, and *Ureaplasma urealyticum*.

Prevalence is estimated as the percentage of *T. vaginalis*-positive women out of the total of screened patients during a given period (pre-COVID-19 and COVID-19); these estimates are accompanied by their 95% confidence intervals (95% CI). The odds ratio (OR) was computed using prevalence data (COVID-19 vs. pre-COVID-19) to test the influence of the pandemic on prevalence trends.

On the other hand, descriptive data of *T. vaginalis* infected women are presented as mean (standard deviation, SD) for quantitative variables (variable “patient age” after having checked its normality using the Kolmogorov–Smirnov test) or number (percentage) for categorical variables (“nationality”, “pregnancy”, “symptomatology”, “inflammation”, and “co-infection”). These demographic and clinical characteristics of infected patients were compared between periods, pre-COVID-19 and COVID-19, using the Student *t*-test for independent samples in the case of quantitative variables (this test determines if there is a significant difference between the means of two groups) and the Pearson Chi-squared test or Fisher exact test for categorical variables (both tests determine if there is a significant association between two categorical variables). A *p*-value below 0.05 was considered statistically significant. Data were analyzed using SPSS version 20 (IBM Inc., Chicago, IL, USA).

### 2.5. Ethical Statement

The study protocol was carried out according to the Declaration of Helsinki and was conducted in accordance with applicable ethical norms and regulations. The research project was approved by the Ethics Committee for Research of the Hospital Universitario Puerta de Hierro (CEIm), and granted permission (Acta n° 21.17). The CEIm approved access to medical records of patients positive for *T. vaginalis*. The data have been managed in accordance with the Organic Law 3/2018 of December 5 on the Protection of Personal Data and Guarantee of Digital Rights, and remain confidential. Personal data were anonymized to protect the privacy of the participants. The compiled data are under the control of the corresponding author on UCM computers with an identification code for access.

## 3. Results

Before describing the differences between the demographic or clinical characteristics of patients before and during the pandemic, it is important to highlight how trichomoniasis has presented in Spain during these years. Out of the total of diagnosed patients over the four years (145), only six were men (4.1%), and therefore they were excluded from the analysis set. Regarding the demographic characteristics of the patients, the mean age was 40.3 years, with 63% of the patients being over 35 years old; 57% were of European nationality (mainly Spanish), followed by 42% of patients who were from Central or South America. As for the clinical analysis, 9.4% of the women were pregnant.

Concerning the clinical characteristics, 5% of the patients were asymptomatic, all of them corresponding to patients diagnosed from 2018 and 2019. The most common signs/symptoms, when present, were leukorrhea (particularly in the pandemic period) and pruritus, while erythema, bleeding, or fever were rarely mentioned.

The vaginal microbiota was also studied. It was considered normal in cases where lactobacilli predominated, altered when a mixture of lactobacilli and anaerobic bacteria was found, and bacterial vaginosis when anaerobic bacteria predominated. Excluding the ten unknown samples, the most common finding was altered microbiota (56.6%), although 38.0% had normal flora. Moreover, 4.7% had candidiasis, and only 0.8% showed bacterial vaginosis.

The inflammation parameters were classified into three groups based on the frequency of leukocytes as a sign of genital inflammation (absent, few, or frequent). In this case, balanced results were observed among patients who showed no leukocytes in the fresh examination (41.0%), and those in whom abundant leukocytes were observed (38.1%).

Over the pre-pandemic period from January 2018 to December 2019, 95 women were diagnosed with trichomoniasis from a total of 8253 screened patients. The percentage of *T. vaginalis*-positive women was 1.15% (95% CI: 0.94–1.41). In contrast, during the pandemic, considering cases from January 2020 to December 2021, 44 women were diagnosed with trichomoniasis from a total of 5728 screened patients. The prevalence in this period was 0.77% (95% CI: 0.57–1.03); therefore, the odds of *T. vaginalis* infection significantly decreased in the pandemic period (Odds Ratio, OR: 0.66, 95% CI: 0.46–0.95; *p* = 0.025).

The average number of samples analyzed was 397 in 2018, 419 in 2019, 248 in 2020 (with clear differences between January–February and the following months), and 299 in 2021. Likewise, more than 76% of the *T. vaginalis* cases in 2021 were diagnosed during the second semester.

Demographic and clinical characteristics of *T. vaginalis* patients before and during the COVID-19 pandemic were similar (Table 1). The mean age of the patients was stable, at 40 years old. More than half of the women diagnosed during the pandemic period were of Spanish nationality, compared to 37.2% in the former period, but this difference was not statistically significant (*p* = 0.110), nor did they differ with respect to pregnancy rate.

Analyzing reports containing data of signs and symptoms (data available for 71 out of the 95 pre-pandemic patients, and for 26 out of the 44 pandemic patients), 93.0% and 100% were symptomatic, respectively (Table 1). No differences were observed in symptomatology before vs. during COVID-19 (*p* = 0.320, Table 1), nor in leucocytes analyses (*p* = 0.608, Table 1). However, it should be noted that 59.1% (26/44) of patients diagnosed during the pandemic period were attended by the Urgent Care Services (data not available for the previous period). There were no differences found in terms of microbiota status before and during the pandemic, although a trend towards more altered flora during the pandemic is observed (57% vs. 47%).

Regarding signs and symptoms recorded in pregnant patients, half of them (5/10) were symptomatic in the pre-pandemic period (one case of vaginal bleeding with hypochondrial pain, another with dysuria, the third one with fever, and two cases with leukorrhea). During the pandemic, three pregnant women were diagnosed: one patient with premature rupture of membranes (week 34) with *Ureaplasma* co-infection, a second pregnant case with pelvic inflammatory disease (PID), and a third case with no recorded specific data about symptoms, despite being listed as symptomatic in the clinical history.

During the pandemic period there was no change in protocols that lead to differences in co-testing. The screening and presence of other STIs, or *M. hominis* and *U. urealyticum*, are shown in Table 2 for every patient (95 + 44), and in Table 3 for pregnant patients (10 + 3) only; where comparison between periods was possible, no differences in positivity rates were observed (*p* > 0.05). Noteworthy, there were four cases of pelvic inflammatory disease (PID) in total, one before COVID-19 and three during the pandemic, and one co-infected with *C. trachomatis*. Moreover, one case of high-risk papillomavirus was seen in a patient, and four cases had genital herpes. Except for *N. gonorrhoeae*, co-infection screening was performed in a remarkable low number of patients (Table 3).

## 4. Discussion

Before the pandemic (2018 and 2019), the prevalence of trichomoniasis in the current study was 1.15%. Comparing these results with a study conducted in the same tertiary hospital of Spain [7], a significant upward trend compared to previous years is observed, rising from 0.51% in 2016 [7] to 1.09% in 2019. These data underscore a concerning escalation in the prevalence of trichomoniasis. In comparison with data from other regions of the country, it is slightly lower than the 2.4% reported by Carrillo-Ávila et al. (2017) in the Southeast of Spain, but almost identical to the 1% detected by Sorlózano-Puerto et al. (2018) in a tertiary hospital of the same region [4,5]. Our results are also in the same range as those described by neighboring countries like Portugal (1.0%), France (1.7%), or the Netherlands (1.4%) [31,32,33].

During the pandemic, data from this study show that the prevalence was reduced to 0.77%. Sexual relationships were affected by factors such as stress, reduced libido, fear of pregnancy, social distancing, or the presence of children at home [28]. Diagnoses of STIs were impacted, especially those infections that are asymptomatic and rely on screenings for diagnosis and treatment [34]. According to our data, a significantly lower number of vaginal samples were analyzed in the tertiary hospital in 2020–2021 (6565), a third less than in 2018–2019.

According to the CDC, the impact of the pandemic continued into 2021, and therefore, studies conducted during this period remain significantly affected by it [34]. In our study, a significant reduction in trichomoniasis cases in 2020 and 2021 stands out, with the last quarter of 2021 accounting for 57% of the cases for that year, which could be a sign of recovery from the pre-pandemic levels. Our results are consistent with other studies performed in Catalonia and Valencia (Spain) [35,36]. The first research group found a significant reduction in STI diagnoses (i.e., chlamydiasis, gonorrhoea, syphilis, and lymphogranuloma venereum) during the lockdown period and almost five months later [35]. The research conducted by Casanova-Esquembre et al. reports a clear negative correlation between positive cases of SARS-CoV-2 and STIs, with a sharp decline in venereal infections during the first wave of SARS-CoV-2 in March 2020 [36]. Interestingly, and in line with our data, they also found that by the end of 2021, the number of STI-positive samples had returned to a level similar to those seen in pre-pandemic years. This reduction of STI cases during 2020 was also observed in Cuba, Italy, the USA, and Spain [29,35,37,38]. Therefore, it would be interesting to see how the prevalence evolves during the next years, when normality has been completely established.

On the other hand, before the pandemic, only 7% of cases analyzed in this study were asymptomatic, compared to 25–50% of infected women without symptoms reported by other studies and reviews [39,40]. In contrast, all patients diagnosed during the pandemic were symptomatic (among the patients with reports). In this sense, we highlight the absence of a routine screening of this STI in healthy women during gynecological revisions or, even more importantly, during pregnancy, which would explain the scarce number of cases diagnosed in asymptomatic women. In other words, the health system is primarily detecting symptomatic patients, as they are likely the individuals who seek medical attention due to their discomfort. Considering the high number of asymptomatic patients with this STI [40], the real prevalence in this population will probably be higher, and therefore its control requires the diagnosis of any carrier; nevertheless, asymptomatic patients may have not been diagnosed because nowadays trichomoniasis is not considered as significant as other STIs, like syphilis or gonorrhoea. This fact has already been warned by other authors [41,42] and a change in the perception of the seriousness of this infection is necessary.

It is important to highlight that almost one third of the patients were attended by the Urgent Care Services during 2020–2021. This could be indicative of how healthcare services were disrupted by the focus on SARS-CoV-2. The pressure and restructuring of triage procedures to which emergency departments and hospitals were subjected [43] would explain why there were 18 patients during the period of 2020–2021 for whom no data were included in their medical records regarding symptoms.

In the context of vaginal dysbiosis, *T. vaginalis* and its endobionts make up a singular microbial entity that can lead to severe sequelae, including preterm delivery, and the acquisition and transmission of HIV [44]. Although both *M. hominis* and *U. urealyticum* are considered commensal of the genital tract, in pregnant women their presence is closely linked to undesirable pregnancy outcomes [45,46]. In this context, it is important to note the percentage of pregnant women who were also positive to *U. urealyticum* (63%) and *M. hominis* (29%), as shown in Table 3, for the increased potential risk of adverse outcomes [47]. Considering the percentage of co-infections with *M. hominis* and *U. urealyticum* in patients with trichomoniasis, and the consequences of having these pathogens, especially during pregnancy, it might be advisable to recommend the diagnosis of the three microorganisms in cases of suspicion of any of them. Although the Center for Disease Control and Prevention (CDC) recommends screening for women at high risk for infection [48] in their STI Treatment Guidelines, this practice is not carried out. Interestingly, these Guidelines do not show any recommendation for men, who are the main carriers of the disease [49], nor for pregnant women. In this study we detected pregnant women positive to *T. vaginalis* and mycoplasmas with clinical complications such as light bleeding or hypochondrial pain. Therefore, it seems appropriate to include this STI in routine screening during pregnancy and to evaluate whether these isolates harbor mycoplasmas.

It is surprising that even when trichomoniasis is the most common non-viral STI worldwide, it is still a parasitic neglected infection [50,51]. It might not cause severe cases as often as other curable STIs like *N. gonorrhoeae* or *C. trachomatis*, but when it does, there is only one family of drugs available and it is sometimes inefficient or controversial, like in pregnancy or breastfeeding [52,53]. Recently, different studies have warned of the risk of treating trichomoniasis in pregnant women due to the possibility of release of endobionts (TVV or mycoplasmas) that could infect fetal membranes or trigger proinflammatory responses with the consequent risk during pregnancy [54,55]. The results presented herein warn of the increase in the number of pregnant women infected with *T. vaginalis* and how in some patients the parasite cohabitates with *Mycoplasma* or *Ureaplasma*. This study also warns of the lack of protocols related to screening for other STIs when *T. vaginalis* is diagnosed. As previously indicated, this parasite increases the risk of acquiring other pathogens after sexual intercourse, and co-infections are common [56,57,58]; some of them (co-infection with high-risk HPV) promote the development of cervical cancer [11]. Moreover, if trichomoniasis is not diagnosed, persistent infection may be established, inducing a chronic inflammatory microenvironment that might contribute to prostate cancer progression [10]. In summary, the routine screening of *T. vaginalis* may be used as a marker of risk sexual behavior and to consider in screening for cancer. Indeed, after the pandemic, more and more healthcare organizations are encouraging clinics to actively request STI co-testing when a patient is positive for one, especially HIV and *C. trachomatis* [15].

Limitations of our study may arise from aggregating the data into two two-year periods, impairing the detection of existing trends of the disease before the pandemic (and afterwards as well). However, our main goal was to determine the COVID-19 pandemic impact in the epidemiology of the disease and this grouping ensured sufficient statistical power in the comparative analysis of the demographic and clinical characteristics of patients affected by *T. vaginalis*, where the limited frequency of patients per year (especially in the pandemic period) would have compromised this analysis and its conclusions.

Other study limitations include the absence of data (signs and symptoms) on certain patients infected with *T. vaginalis* in their medical records and the fact that the origin (Primary Care, Urgent Care Service, etc.) of some vaginal samples analyzed during the pre-pandemic period was unknown. Furthermore, it was not possible to compare with the clinical demographic characteristics of pregnant women who did not have trichomoniasis, as the data provided by the hospital and approved by the Ethics Committee only allowed access to the medical records of patients positive for this infection. In addition, the samples of pregnant patients were small, making it more difficult to find significant differences when performing statistical analyses. Moreover, it should be noted that there are no screening programs for this infection, so in most cases, the patients diagnosed are those who attend the hospital with symptoms suggestive of trichomoniasis. Therefore, the rate of symptomatic cases is higher than usual, and the prevalence may be underestimated.

Although active screening for trichomoniasis was conducted from the Microbiology Service of the tertiary hospital among all the vaginal swabs received in both periods, this is not the standard of care. Microbiology services of Spain usually look for *T. vaginalis* isolation in vaginal swabs, using traditional techniques like direct microscopy or culture following the protocols described by the Spanish Society of the Spanish Society of Infectious Diseases and Clinical Microbiology [30]. However, even when the microscopic methods are often set, it does not have high sensitivity (40–70%), depending on many factors [59]. Fortunately, many Microbiology Services are setting biomolecular techniques with high sensitivity and specificity for STI infections, and multiplex PCR is becoming an interesting alternative to detect more than one genitourinary pathogen simultaneously from one sample [60,61]. However, trichomoniasis is only diagnosed with molecular tools if the clinician specifically requests to look for this parasite.

Based on the above, it would be interesting to carry out innovative studies on an international scale to consolidate a larger set of samples, thus facilitating the drawing of more robust conclusions. It would also be advisable to carry out a comprehensive analysis of STIs, including the neglected *T. vaginalis*. In this context, some hospitals are carrying out general STI screening in certain populations to determine the true impact of these pathologies in order to establish new protocols. Likewise, it would be of great interest to assess *M. hominis* in women planning pregnancy in order to treat patients and avoid the risk associated with both pathogens in pregnant women. Moreover, in pregnant women diagnosed with trichomoniasis, endobionts should be evaluated due to the risk of “bacterial pinata” [54,55,62] to individualize the therapeutic decision.

## 5. Conclusions

The present research aimed to study how the pandemic has impacted the diagnosis of trichomoniasis in a tertiary hospital of Madrid, Spain. During the COVID-19 pandemic, the number of vaginal samples analyzed, and consequently the trichomoniasis prevalence, was reduced. It is noteworthy that some of the patients were recurring at the hospital through Urgent Care Services or with complications, aspects which should be considered when calculating the direct and indirect costs of this infection. Therefore, in this scenario, it is important to continue studying the effect of *T. vaginalis* during pregnancy and evaluating the presence of endobionts due to their potential clinical effects, as well as to include the diagnosis of this parasite and co-infections in routine protocols with special emphasis during pregnancy. Moreover, the presence of *T. vaginalis* may be an interesting marker of sexual risk behavior that should be considered by clinicians to explore the presence of other sexually transmitted pathogens.

## Figures and Tables

**Table 1:** Demographic and clinical characteristics of *T. vaginalis* infected women in both periods of study.

	2018–2019	2020–2021	*p*-Value
Number of patients	95	44	
Patient’s age (years old; mean ± SD)	40.3 (±11.5)	40.2 (±9.8)	0.960
Nationality			0.110
Spanish	35/94 (37.2)	24/44 (54.5)	
South/Central Americans	42/94 (44.7)	12/44 (27.3)	
Others	17/94 (18.1)	8/44 (18.2)	
Non-pregnant	85 (89.5)	41 (93.2)	0.755
Pregnant	10 (10.5)	3 (6.8)	
Asymptomatic	5/71 (7.0)	0/26 (0.0)	0.320
Symptomatic	66/71 (93.0)	26/26 (100)	
Signs and Symptoms ^1^:			
Leukorrhea	30 (45.5)	22 (84.6)	
Pruritus	18 (27.3)	10 (38.5)	
Vaginitis/vulvitis	19 (28.8)	0 (0.0)	
Dysuria	5 (7.6)	4 (15.4)	
Inflammation parameters			0.608
No leukocytes	40/91 (43.9)	15/43 (34.9)	
Few leukocytes	18/91 (19.8)	10/43 (23.2)	
Frequent leukocytes	33/91 (36.3)	18/43 (41.9)	

Data are shown as *n* (% of total with data), except where otherwise indicated. Denominator specified in case of missing data for a characteristic. ^1^ Signs and symptoms data were available for 66 (93.0%) symptomatic patients diagnosed in 2018–2019 and for every symptomatic patient diagnosed in 2020–2021; signs and symptoms were not mutually exclusive (therefore, n and % correspond to the number and proportion of symptomatic patients in whom this sign/symptom, alone or in combination with others, was present). SD: Standard deviation.

**Table 2:** Performance and results of women co-infected with *T. vaginalis* and other sexually transmitted pathogens (95 patients in 2018–2019 and 44 patients in 2020–2021); positive rate comparison between periods. *M. genitalium* is not included because there was no PCR available for its detection.

Pathogen	Performed (%)		Positive (%) among Performed		*p*-Value
	2018–2019	2020–2021	2018–2019	2020–2021	
*Neisseria gonorrhoeae*	73 (76.8)	43 (97.7)	0/73 (0.0)	0/43 (0.0)	--
*Chlamydia trachomatis*	25 (26.3)	21 (47.7)	2/25 (8.0)	4/21 (19.0)	0.390
Human Immunodeficiency Virus	34 (35.8)	13 (29.5)	0/34 (0.0)	0/13 (0.0)	--
*Treponema pallidum*	32 (33.7)	13 (29.5)	1/32 (3.1)	0/13 (0.0)	1.000
*Mycoplasma hominis*	23 (24.2)	0 (0.0)	4/23 (17.4)	--	--
*Ureaplasma urealyticum*	23 (24.2)	1 (2.3)	6/23 (26.1)	1/1 (100)	0.292

Data are shown as *n* (% of total with data).

**Table 3:** Performance and results of STI co-infection screening in *T. vaginalis* infected pregnant women (ten pregnant patients in 2018–2019 and three pregnant patients in 2020–2021).

Pathogen	Performed (%)		Positive (%) among Performed	
	2018–2019	2020–2021	2018–2019	2020–2021
*Neisseria gonorrhoeae*	7 (70.0)	3 (100)	0/7 (0.0)	0/3 (0.0)
*Chlamydia trachomatis*	4 (40.0)	2 (66.7)	0/4 (0.0)	0/2 (0.0)
Human Immunodeficiency Virus	6 (60.0)	1 (33.3)	0/6 (0.0)	0/1 (0.0)
*Treponema pallidum*	6 (60.0)	1 (33.3)	1/6 (16.7)	0/1 (0.0)
*Mycoplasma hominis*	3 (30.0)	0 (0.0)	1/3 (33.3)	--
*Ureaplasma urealyticum*	3 (30.0)	1 (33.3)	2/3 (66.7)	1/1 (100)

Data are shown as *n* (% of total with data). *p*-values not included due to the following: impossibility of comparison (zero proportion) or lack of significance (*p* = 1.000) where analysis was possible (*T. pallidum* and *U. urealyticum rows*).

## Data Availability

The data presented in this study are available from the corresponding author upon request. The data are not publicly available due to the privacy of the participants.
